# Eco-evolutionary dynamics of dispersal in spatially heterogeneous environments

**DOI:** 10.1111/j.1461-0248.2011.01671.x

**Published:** 2011-10

**Authors:** Ilkka Hanski, Tommi Mononen

**Affiliations:** Department of Biosciences, University of HelsinkiFI-00014 Helsinki, Finland

**Keywords:** Extinction-colonisation dynamics, local adaptation, habitat loss and fragmentation, phosphoglucose isomerase, *Pgi*, Glanville fritillary, *Melitaea cinxia*

## Abstract

Evolutionary changes in natural populations are often so fast that the evolutionary dynamics may influence ecological population dynamics and *vice versa*. Here we construct an eco-evolutionary model for dispersal by combining a stochastic patch occupancy metapopulation model with a model for changes in the frequency of fast-dispersing individuals in local populations. We test the model using data on allelic variation in the gene phosphoglucose isomerase (*Pgi*), which is strongly associated with dispersal rate in the Glanville fritillary butterfly. Population-specific measures of immigration and extinction rates and the frequency of fast-dispersing individuals among the immigrants explained 40% of spatial variation in *Pgi* allele frequency among 97 local populations. The model clarifies the roles of founder events and gene flow in dispersal evolution and resolves a controversy in the literature about the consequences of habitat loss and fragmentation on the evolution of dispersal.

## Introduction

Population biologists are increasingly concluding that microevolutionary changes are often so fast in natural populations (Thompson 1998; Hendry & Kinnison 1999; Saccheri & Hanski 2006) that the evolutionary dynamics may influence ecological population dynamics and *vice versa*. Such coupled ecological and evolutionary dynamics, or eco-evolutionary dynamics for short (Pelletier *et al.* 2009), have been analysed with models in the context of, for instance, the dynamics of species’ range boundaries (Kirkpatrick & Barton 1997), the evolution of species’ niches (Kawecki 1995) and predator-prey dynamics (Abrams & Matsuda 1997). Empirical studies are less common, and even the ones that have been published under the banner of eco-evolutionary dynamics are mostly concerned with phenotypic or genotypic effects on population dynamics (Hairston *et al.* 2005; Hanski & Saccheri 2006; Ezard *et al.* 2009) rather than with reciprocal effects between ecological and evolutionary dynamics (Sinervo *et al.* 2000; Zheng *et al.* 2009).

Dispersal is a good candidate for a process that might exhibit reciprocal eco-evolutionary dynamics in many species and environments. Dispersal clearly influences ecological spatial dynamics as well as the dynamics of local adaptation via founder events, gene flow and life history trade-offs (for the latter see e.g. Zera & Denno 1997). Dispersal may evolve fast especially in colonising species and in metapopulations inhabiting heterogeneous environments (reviewed by Reznick & Ghalambor 2001). Thus dispersal may often exhibit complex eco-evolutionary dynamics in which demographic dynamics influence microevolutionary dynamics and *vice versa*.

Much of the research on the evolution of dispersal has been conducted on species exhibiting discrete variation (polymorphism) in dispersal capacity (Roff & Fairbairn 1991), because such species offer an important practical advantage for research: distinguishing between fast-dispersing and slow-dispersing individuals is easy. Extreme examples include insect species in which some individuals are wingless and hence flightless, whereas others have functional wings and disperse long distances (Zera & Denno 1997). Similarly, many plant species have heavy, poorly dispersing seeds as well as light seeds with morphological structures facilitating long-distance dispersal (Venable 1979). More subtle cases are exemplified by the Glanville fritillary butterfly (*Melitaea cinxia* L.), in which a single nucleotide polymorphism in the phosphoglucose isomerase (*Pgi*) gene (Orsini *et al.* 2009) is associated with significant differences in flight metabolic rate (Niitepõld 2010) and dispersal rate in the field (Niitepõld *et al.* 2009).

It is common knowledge that, in species exhibiting dispersal polymorphism, the more dispersive phenotype predominates in unstable habitats and populations, while the less dispersive phenotype is common in stable habitats and populations (Southwood 1962), in support of the theoretical prediction that temporal variation in environmental conditions selects for dispersal (Comins *et al.* 1980). It is less clear how, and why, dispersal rate varies spatially and temporally in heterogeneous environments. For instance, habitat loss and fragmentation may either decrease or increase dispersal (Ronce & Olivieri 2004; Hanski 2005), most likely depending on the relative strengths of the many factors that influence the evolution of dispersal, including habitat heterogeneity and perturbations, inbreeding, competition with related and non-related individuals, and the cost of dispersal (for reviews see Clobert *et al.* 2001; Ronce 2007).

In this paper, we modify an eco-evolutionary metapopulation model described by Hanski *et al.* (2011) to analyse dispersal polymorphism in heterogeneous environments. The model combines a stochastic patch occupancy metapopulation model (Hanski 1998a) with a model of local adaptation describing changes in the mean phenotype in local populations. Here, the mean phenotype is the frequency of fast-dispersing individuals in a local population. The model is constructed at the level of local populations rather than individuals, which prevents the analysis of many general questions about the evolution of dispersal. Instead, our model is aimed at analysing spatial variation in the long-term frequency of fast-dispersing individuals among local populations in a network of habitat patches with an explicit spatial structure. We test the model with data on spatial variation in *Pgi* allele frequency in the Glanville fritillary across a large patch network.

## Model, Material and Methods

### Model construction

The ecological dynamics are described with a stochastic patch occupancy model (Hanski 1998a), which specifies the rates of colonisation and extinction in a network of *n* patches. The colonisation rate of patch *i* at time *t,* if unoccupied, is given by 
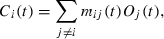
(1) where *O*_*j*_(*t*) denotes the occupancy state (1 or 0) of patch *j* at time *t* and *m*_*ij*_(*t*) gives the contribution of population *j* to the colonisation of patch *i.* The extinction rate of population *i* at time *t* is denoted by*E*_*i*_(*t*). Below, we make assumptions about how the network structure influences the values of *m*_*ij*_(*t*) and *E*_*i*_(*t*) to complete the ecological part of the model. The evolutionary part specifies how the colonisation and extinction rates depend on the mean dispersal phenotype in local populations.

#### Model for dispersal polymorphism as a local adaptation

We assume that there are two kinds of individuals with fixed rates of dispersal: the slow-dispersing individuals emigrate with rate *ε* and the fast-dispersing ones with rate Δ*ε*, where Δ > 1. The mean dispersal phenotype 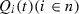 is defined as the frequency of fast-dispersing individuals in population *i* at time *t.* When a new population becomes established in patch *i,* the value of *Q*_*i*_ is defined as the weighted average of the mean phenotypes of the surrounding populations from which the emigrants that contributed to the colonisation departed. We thus assume the migrant pool model of colonisation (Slatkin 1977), with *m*_*ij*_ giving the weight of population *j.* The mean phenotype of population *i* at colonisation is then given by 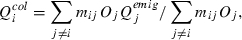
(2) where 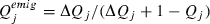 is the mean phenotype of emigrants departing from population *j.*

Following colonisation, the value of *Q*_*i*_ changes according to the following equation, which accounts for the effects of emigration, local selection and immigration on the rate of change in *Q*_*i*_
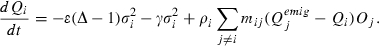
(3) The first term describes the effect of emigration and is derived as follows. Consider that there are *N*_*s*_ and *N*_*f*_ slow-dispersing and fast-dispersing individuals in the population, respectively. Given that the numbers of emigrants are proportional to *ε N*_*s*_ and Δ*ε N*_*f*_, the rate of change in the ratio *Q*_*i*_ *= N*_*f*_/(*N*_*s*_ + *N*_*f*_) due to emigration can be calculated as 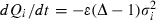, where 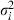 is short for *Q*_*i*_ (1–*Q*_*i*_). The next term describes the effect of local selection. The slow-dispersing individuals may have higher fitness locally than the fast-dispersing individuals due to life history trade-offs (Zera & Denno 1997). Based on an analogous argument to that for emigration, the term 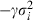 gives the rate of change in *Q*_*i*_ due to local selection, with parameter *γ* giving the strength of selection. Finally, the third term in Eq. (3) gives the rate of change in *Q*_*i*_ due to immigration, which may either increase or decrease *Q*_*i*_ depending on the mean phenotypes of emigrants originating from the different source populations. The term describing the effect of immigration is the same as in Hanski *et al.* (2011), whereas the first two terms in Eq. (3) are specific to the present model of dispersal polymorphism. Parameter *ρ*_*i*_ gives the proportionality between immigration (*ρ*_*i*_*C*_*i*_) and colonisation rates (*C*_*i*_) (explained further below). Note that Eq. (3) specifies a deterministic rate of change in *Q*_*i*_ and thus the model ignores drift.

#### Model for extinction rate

We assume that local population dynamics obey the ceiling model described by Lande (1993). In this model, the expected time to population extinction starting at *K,* the population ceiling (carrying capacity), is given by 
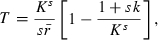
(4) where 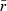 and *v* are the average population growth rate and its variance, respectively, 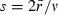 and *k*= ln *K*. Ignoring the transient from the carrying capacity to the quasi-stationary state, we can convert the mean time to extinction in population *i* to extinction rate (probability of extinction per unit time) as 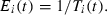
(5)

Though we do not model local dynamics explicitly, the ceiling model is helpful in allowing us to specify how the environment and local adaptation influence the risk of extinction. We assume that *K*_*i*_ is proportional to *A*_*i,*_ and thus the risk of extinction is inversely related to patch area, which is commonly observed (Hanski 2005). Extinction risk increases with decreasing strength of environmental stochasticity, which is measured by *s* (Lande 1993; Hanski 1998b).

To model the effect of local adaptation on extinction risk, we first write an equation for the growth rate of population *i* as (Lande & Shannon 1996) 
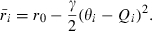
(6)

By the assumptions of the present model, the optimal mean phenotype *θ*_*i*_ is zero in every population, meaning that a population consisting of slow-dispersing individuals only (*Q*_*i*_ = 0) has the maximal growth rate given by *r*_*0*_. The second term represents the demographic cost of maladaptation (large *Q*_*i*_), which decreases population growth rate and thereby increases the extinction rate (Eq. 4).

#### Model of colonisation rate

The colonisation rate is given by Eq. (1). We are now ready to define the contribution of the source population *j* to the colonisation of patch *i* as 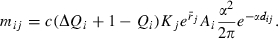
(7)

This equation is the same as in Hanski *et al.* (2011) except for the term Δ*Q*_*i*_ + 1 – *Q*_*i*_, which takes into account the difference in the emigration rates of the two kinds of individuals. Briefly, *c* scales the overall rate of dispersal, including the effect of dispersal mortality (decreases *c*); immigration to patch *i* increases with patch area *A*_*i*_; the contribution of patch *j* increases with its carrying capacity *K*_*j*_, assumed to be proportional to patch area, and with its growth rate 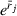; and the contribution of population *j* to immigration to patch *i* increases with decreasing distance *d*_*ij*_ between the two patches (exponential dispersal kernel with parameter *α*). Depending on the biology of particular species, one may want to change some of these assumptions.

#### Deterministic approximation of the stochastic model

It is straightforward to simulate the stochastic extinction-colonisation model defined by Eqs. (1) and (5), using Eq. (3) to calculate the deterministic rate of change in the mean phenotype in the occupied patches. A drawback of simulations is that they are relatively slow for large networks and it is difficult to arrive at general conclusions. We therefore use a deterministic approximation of the quasi-stationary state of the stochastic model to obtain insight into model predictions and to apply it to empirical data. The deterministic approximation is given by the equations (see Hanski *et al.* 2011) 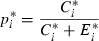
(8)
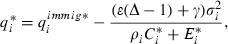
(9) where 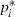 is the probability of patch *i* being occupied in the long course of time and 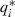 is the corresponding frequency of fast-dispersing individuals conditional on occupancy. The asterisk (*) denotes an equilibrium value. We use *q* for the frequency of fast-dispersing individuals in the approximation to distinguish it from the corresponding variable in the stochastic model (*Q*). The variable 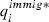 is the equilibrium dispersal morph frequency among the immigrants arriving at patch *i*, defined as the weighted average of the 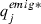 values 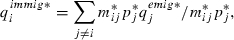
(10) with 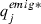 given by 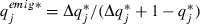. Concerning the proportionality between immigration to and colonisation of patch *i,* we assume that *ρ*_*i*_ = *ρ*/*A*_*i*_, and thus immigration is measured in terms of the numbers of immigrants in relation to the size of the resident population (measured by patch area).

**Figure 1:**
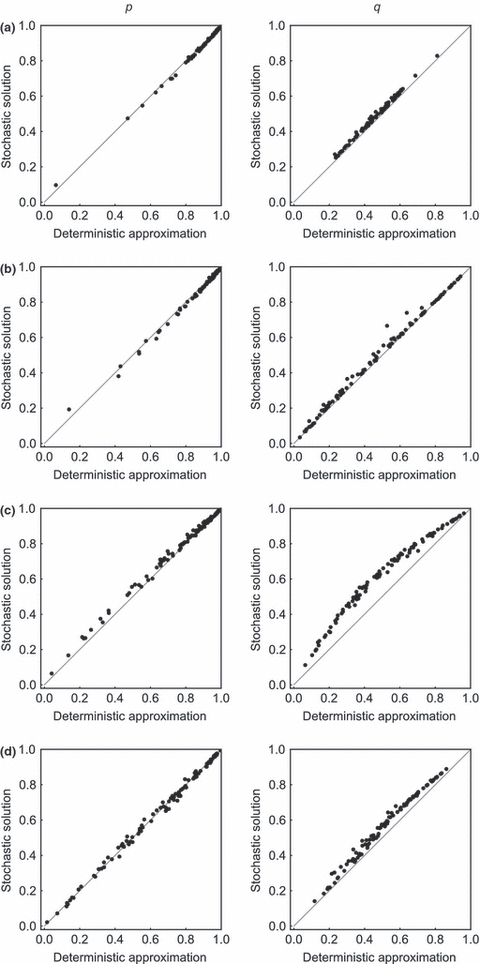
Comparison between patch-specific incidences of occupancy (*p*_i_*; left panels) and mean dispersal phenotypes (*q*_i_*; right panels) in the quasi-stationary state of the stochastic model (vertical axis) and its deterministic approximation (horizontal axis) in a heterogeneous network of 100 habitat patches. Patch areas are log-normally distributed, with mean of 2.0 and standard deviation of 0.5, and the patches have random spatial locations within a square area of 10 by 10 units (note that patch areas are not measured in the same unit). Parameter values: (a) *α*= 0.5, *γ*= 0.75, *c*=0.0065, *ε*= 0.001, *ρ*= 2.5, Δ = 5; (b) *α*= 2.0, *γ*= 0.75, *c*=0.0039, *ε*= 0.001, *ρ*= 2.5, Δ = 5; (c) *α*= 1.5, *γ*= 0.0, *c*=0.0009, *ε*= 0.05, *ρ*= 2.5, Δ = 5; and (d) *α*= 1.5, *γ*= 0.0, *c*=0.00085, *ε*= 0.05, *ρ*= 2.5, Δ = 2. In all panels, *r*_*0*_ = 1 and *v*=1.

Hanski *et al.* (2011) present the justification for the approximation in the case of the general eco-evolutionary model, which we have modified here to model the dynamics of dispersal polymorphism. The present model has always a unique quasi-stationary state. Figure 1 compares for a range of parameter values the deterministic approximation with the quasi-stationary state of the stochastic model in a heterogeneous network of 100 habitat patches. The approximation is generally very good; it is worst when Δ is large and *γ* is small (Fig. 1).

### Empirical data on *Pgi* allele frequency

The model that we have constructed can be tested with data on spatial variation in the mean dispersal phenotype among local populations in a metapopulation. We have tested the model (Eq. 9) with data for the Glanville fritillary butterfly, which has a non-synonymous SNP in the coding region of the gene phosphoglucose isomerase (*Pgi_111*; Orsini *et al.* 2009) that is strongly associated with mobility as measured by tracking free-flying butterflies in the field with harmonic radar (Niitepõld *et al.* 2009).

We have genotyped a large material sampled from a network of *c.* 4000 small dry meadows (average area 0.15 ha) within an area of 50 by 70 km in the Åland Islands in southwestern Finland (Hanski 1999; Nieminen *et al.* 2004). The genetic sample was obtained in 2002, when one individual from each larval family group (*n*=2052) in each existing local population (*n*=518) was sampled and genotyped for *Pgi_111* (the larvae live gregariously in groups of full sibs; Hanski 1999). As the patch network has been surveyed annually since the early 1990s (Nieminen *et al.* 2004), we know which of the meadows had been occupied in the previous summer (old populations). The ones that were not occupied in 2001 must have become colonised by dispersing females in the summer 2002 (new populations).

Over a broad range of ambient temperatures, the AC heterozygotes in *Pgi_111* fly roughly twice the distance in a given time than the AA homozygotes (Niitepõld *et al.* 2009). In the Åland Islands, though not everywhere within the geographical range of the Glanville fritillary, the CC homozygotes are very rare (Orsini *et al.* 2009). It appears that most CC homozygotes die at an early stage of development, possibly due to linkage with a lethal mutation in a common haplotype. Molecular evidence indicates long-term balancing selection in *Pgi* (Orsini *et al.* 2009; Wheat *et al.* 2010), and experimental studies show that AC heterozygotes have superior performance to AA homozygotes in most fitness components under conditions that commonly prevail in the field (Hanski & Saccheri 2006; Niitepõld *et al.* 2009; Saastamoinen *et al.* 2009; Zheng *et al.* 2009). Here, we define the mean dispersal phenotype *q*_*i*_ in population *i* as the frequency of the C allele in *Pgi_111*, which is a good measure of *q*_*i*_ given that there are essentially two genotypes in this metapopulation (results were similar when *q*_*i*_ was defined as the pooled frequency of the AC and CC genotypes). In the case of the smallest populations, allele frequencies are greatly affected by genetic drift, which is not included in the model. To reduce the effect of drift on the results, we excluded the smallest populations with *N*<6 individuals genotyped while testing the population-specific model prediction. Additionally, we excluded six small populations in which *q*_*i*_ = 0 and one population in which *q*_*i*_ > 0.7. These values are likely to result from drift and they were outliers in the dataset. The remaining material consists of 97 local populations out of the 518 populations sampled, with a pooled sample size of 1142 individuals. To ascertain that the results were not sensitive to the exact cut-off point we repeated the analysis after excluding populations with either *N*<4 or *N*<10 individuals genotyped, leaving 158 and 46 populations with the pooled sample sizes of 1406 and 781 individuals, respectively.

### Testing model predictions

We cannot estimate the primary model parameters with independent data to calculate the mean dispersal phenotype according to Eq. (9), but we can test the predicted dependence of *q*_*i*_ on measures that approximate patch-specific immigration rate (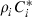), extinction rate (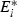) and the equilibrium dispersal morph frequency among the immigrants (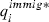). As we have genetic data for one year only, we have to assume that this year is representative of the long-term steady state.

Our surrogate measures of 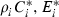 and 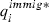, which can be calculated with the empirical data, are as follows. The immigration rate 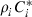 (see Eq. 7) is approximated with a measure of connectivity, 
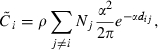
(11) where *N*_*j*_ is the number of larval groups in population *j* in 2002, *d*_*ij*_ is the distance between patches *i* and *j* in km, and *α*= 1 based on empirical data (Hanski 1999). Note that patch area *A*_*i*_ cancels out in Eq. (11). While calculating 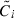 for patch *i,* we included all the 518 local populations in the sum in Eq. (11).

Our surrogate measure of extinction rate is 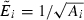, where *A*_*i*_ is the area of patch *i.* This assumes that small habitat patches tend to have small populations with a high rate of extinction, as observed for the Glanville fritillary and many other species (Hanski 2005). Finally, the variable 
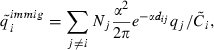
(12) is approximately proportional to 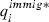 (Eq. 10). The essential difference is that 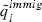 is the weighted average of the *q*_*j*_ values (allele frequencies in the source populations) rather than of the 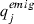 values (allele frequencies among the respective emigrants), which cannot be observed empirically. Therefore, the 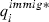 values are systematically greater than the 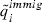 values. We need to specify their relationship below, and we assume that 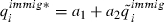, where *a*_*1*_ and *a*_*2*_ are two parameters (both > 0).

The key prediction of the model concerns spatial variation in 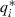, the frequency of fast-dispersing individuals in particular local populations in the patch network. The prediction is given by Eq. (9), which has the term 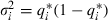 on the right-hand side. Before fitting the equation to the data, we eliminated 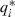 from the right-hand side to obtain a second-order polynomial with the positive root 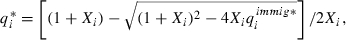
(13) where 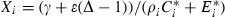. The other root of the polynomial is not biologically feasible (negative population size). While fitting Eq. (13) to the data, the empirical *q*_*i*_ value was the dependent variable and 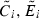 and 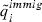 as defined above were the explanatory variables.

To characterise spatial correlation in the *q*_*i*_ values both in the empirical and simulated data, we computed envelopes of Besag's *L*-function for 9999 randomly reshuffled labelings (Illian *et al.* 2008). To produce simulated data that would be comparable with the empirical data we used the stochastic version of the model to simulate patch occupancy and the frequency of fast-dispersing individuals in the sub-set of habitat patches that had been occupied in at least 2 years in 2000–2008 (1037 meadows). The meadows that have been poorly occupied since 2000 are mostly very small and/or have low quality.

## Results

Using the frequency of the C allele in *Pgi_111* as a proxy of the frequency of fast-dispersing individuals in a local population, and the surrogate measures 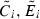 and 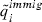 for immigration rate, extinction rate and the mean dispersal phenotype among the immigrants, respectively, we fitted the nonlinear regression model defined by Eq. (13) to the empirical data. The estimated parameter values are given in Table 1. The model-predicted *q*_*i*_ values explain 33% of the variation in the empirical data.

**Table 1:** Spatial variation in the frequency of the *Pgi_111* allele C among 97 local populations in the Glanville fritillary metapopulation. Parameter estimates of the non-linear regression model given by Eq. (13). Linear regression of the empirically measured *q*_*i*_ values against the values predicted by Eq. (13) with the parameter values given in this table explains 23% of the variation in the *q*_*i*_ values. When the regression was weighted with the number of individuals genotyped, to give more weight to the *q*_*i*_ values that are estimated with greater accuracy, adjusted *R*^2^ was increased to 0.33

Parameter	Estimate	SE	95% Confidence intervals	
*a*_*1*_	0.549	0.168	0.215	0.882
*a*_*2*_	0.231	0.057	0.119	0.343
*γ* + *ɛ*(Δ – 1)	0.946	0.686	−0.416	2.308
*ρ*	0.118	0.138	−0.156	0.392

**Figure 2:**
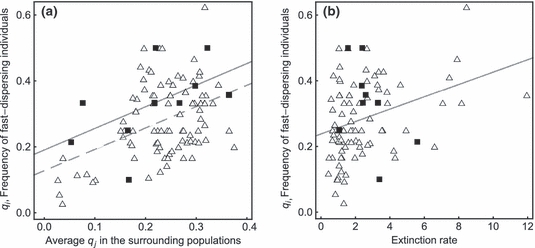
Relationships between the observed frequency of the C allele in *Pgi_111* against (a) the frequency of the C allele in the sources of immigrants (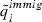) and against (b) the surrogate measure of extinction rate, 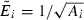, where *A*_*i*_ is patch area. Black squares are for newly-established and open triangles for old populations. In (a) the continuous regression line is for new populations and the broken line is for old populations.

A problem with the above approach is that Eq. (13) is very complex and it may fail because the structural model assumptions do not correspond accurately enough with the real dynamics. We therefore tested, with a simple linear model, the more robust qualititative prediction that the equilibrium allele frequency (*q*_*i*_) increases with 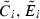 and 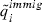. This test ignores the term 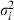 in Eq. (9), which is justified by the empirical values mostly varying within a relatively small range from 0.10 to 0.44 (95% of the values). All three explanatory variables had a significant positive effect in the linear model (Table 2). The interaction between 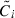 and 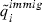
was also significant, due to the effect of 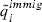 being weaker for high immigration rate. Figure 2 shows the effects of 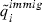 and extinction rate on the frequency of the C allele.

**Table 2:** Step-wise linear regression to explain spatial variation in the frequency of the *Pgi_111* allele C among 97 local populations in the Glanville fritillary metapopulation. The explanatory variables are measures of the frequency of the C allele among the immigrants (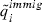), the extinction rate (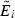) and the immigration rate (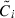) as well as the age of the local population (Age, categorical, new vs. old). To give more weight to populations for which *q*_*i*_ was estimated with greater accuracy, the regression was weighted with *N*, the number of individuals genotyped*. R*^2^ gives the accumulated coefficient of variation in the step-wise regression, the other columns are for the final model. Adjusted *R*^2^ for the full model is 0.40. There was no spatial autocorrelation in the residuals as tested with Moran's I

Step	*R*^2^	Coeff.	SD error	*t*	*P*
Constant	0.00	0.023	0.069	0.33	0.7425
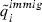	0.27	1.039	0.237	4.38	< 0.0001
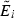	0.34	0.015	0.005	2.94	0.0042
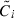	0.37	0.029	0.009	3.04	0.0031
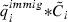	0.40	−0.090	0.038	−2.39	0.0191
Age	0.43	−0.041	0.019	−2.21	0.0294

Finally, we added to the linear model the age of the local population as another explanatory variable. Population age is not a factor in the deterministic equilibrium given by Eq. (9), which averages across populations of different ages, but the model for the rate of change in the mean dispersal phenotype during the life-time of a local population (Eq. 3) typically predicts a decline in the frequency of fast-dispersing individuals following the founder event, because the colonising propagule is biased towards fast-dispersing individuals. Therefore, newly-established populations should have, on average, higher *q*_*i*_ values than old populations. This was indeed the case (Fig. 2a, Table 2). The full model explains 40% of the spatial variation in the *Pgi* allele frequency among the 97 local populations.

The results were similar when we analysed data sets from which populations with < 4 or < 10 individuals genotyped had been excluded, though in these cases the age of the population was not significant (*P*=0.12 and 0.14, respectively). These models explained 28 and 55% of the variation in the *q*_*i*_ values among the 158 and 46 local populations, respectively.

**Figure 3:**
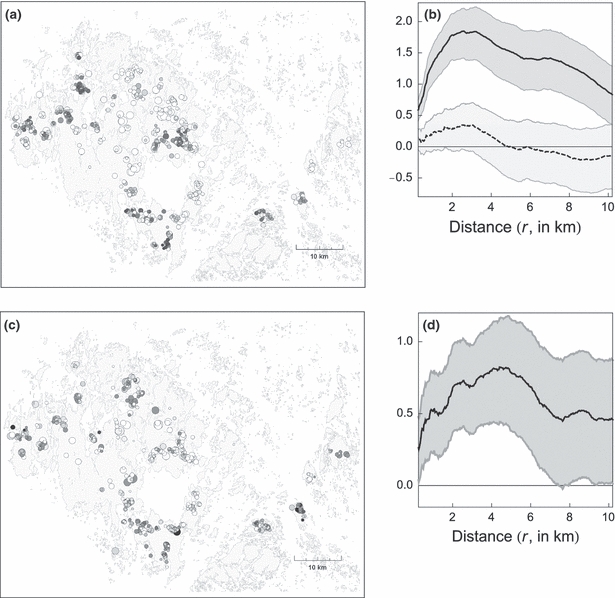
Spatially correlated variation in the frequency of fast-dispersing individuals (*q*_*i*_). (a) A model-predicted quasi-stationary state in terms of the *q*_*i*_ values in the real patch network in the Åland Islands in Finland. The prediction was generated with the stochastic model, which was run for a network of 1037 habitat patches (parameter values *α*= 1, *γ* = 0, *r*_*0*_ = 0.1, *v*=0.5, *ρ* = 0.1, Δ = 5, *ε* = 0.13 and *c*=0.017). Only those patches (*n*=671) that happened to be occupied in the snap-shot that was sampled from the simulation are shown in the figure. The size of the symbol is proportional to patch area, the shading indicates the value of *q*_*i*_. (b) Test of spatial independence of the *q*_*i*_ values by envelopes of Besag's *L*-function. The continuous line gives the mean of the test function for the pattern in (a) with short-range dispersal, the broken line gives the mean for a species with long-range dispersal (*α* = 0.1, *c*=0.09, other parameters as in panel a; *n*=645 occupied patches). When the null line is outside the shaded area, the *q*_*i*_ values for pairs of populations within distance *r* from each other exhibit significant (*P*<0.01) spatial correlation. (c) Empirical result for the Glanville fritillary butterfly, using the frequency of the C allele in *Pgi_111* as a measure of *q*_*i*_ (*n*=518 populations). (d) Test of spatial independence in the empirical data in (c).

To examine the spatial scale of correlation in the model-predicted *q*_*i*_ values we run the stochastic model on the real patch network. For species with short dispersal distances, such as the Glanville fritillary, the model predicts spatially correlated values of *q*_*i*_. The example in Fig. 3a,b was generated by assuming the empirically estimated range of dispersal (*α*= 1; Hanski 1999) and selecting such values for the other parameters that produced a similar number of occupied patches and similar variance of the *q*_*i*_ values than observed in the empirical data. The spatially correlated pattern is robust to changes in the values of the other model parameters as long as dispersal distances are short. In contrast, when dispersal distances are long (*α* small), the *q*_*i*_ values are not spatially correlated (Fig. 3b).

The empirical result on spatial correlation in Fig. 3c,d was calculated for the 2002 sample of 518 populations. The frequency of the C allele in *Pgi_111* was significantly spatially correlated up to several km, and the empirically observed spatial pattern in the *q*_*i*_ values was broadly similar to the model-predicted pattern (Fig. 3). Note that the model in Table 2 accounts for spatial correlation in the *q*_*i*_ values via the term 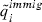, and there was no spatial autocorrelation in the residuals as tested with Moran's I.

## Discussion

Empirical research on the evolution of dispersal has notoriously lagged behind the development of theory and models (Ronce 2007), partly because models typically make very simple assumptions about the spatial structure of the environment and assume global dispersal. In contrast, the present model includes an explicit description of the spatial structure of a patch network and allows for any spatial range of dispersal. This is helpful, because the model then makes testable predictions about how spatial variation in immigration and extinction rates affects population-specific dispersal rates. The cost of including in the model an explicit description of landscape structure is that the model is necessarily simplified in other respects and cannot be used to address all general questions about the evolution of dispersal. For instance, because the present model is constructed at the level of local populations rather than individuals, there is no opportunity to quantify dispersal mortality for individuals, and hence one cannot verify the well-established result that fixed spatial variation in population sizes selects against dispersal (Hastings 1983). Nonetheless, at the qualitative level the present model makes similar predictions than individual-based models, for instance increasing dispersal mortality decreases immigration and colonisation rates and thereby selects against dispersal.

Another limitation of the present model is the focus on the frequency of two pre-defined dispersal phenotypes rather than on the conditions under which dispersal polymorphism will evolve in the first place. Massol *et al.* (2011) have constructed and analysed a model of dispersal evolution focused on kin competition and the cost of dispersal. They show that disruptive selection may lead to dispersal polymorphism when there is sufficient variation in the sizes of local populations and hence in the degree of kin competition, and they refer to the Glanville fritillary metapopulation as a supporting example. The *Pgi* polymorphism which largely underlies variation in dispersal rate in the Glanville fritillary is however not consistent with their model of adaptive dynamics, and it is clear that population turnover due to frequent extinctions is an important factor selecting for dispersal in the Glanville fritillary (the latter process is included in the model of disruptive selection on dispersal by Parvinen 2002).

In the present model, the long-term equilibrium frequency of fast-dispersing individuals in a particular habitat patch is reduced by local selection and biased emigration, while it increases with immigration and the rate of extinction. The first two effects follow directly from model assumptions. As these effects operate in the same manner in the same direction in the compound term in Eq. (9), only one of them is needed to maintain dispersal polymorphism. Thus dispersal polymorphism may be maintained by the cost incurred by high emigration rate to fast-dispersing individuals even in the absence of any other life history trade-offs (see also Cohen & Motro 1989). In the Glanville fritillary, there is no obvious dispersal-fecundity trade-off, but high emigration rate of fast-dispersing individuals, apart from possibly increasing mortality, decreases the time that individuals spend in habitat patches and hence the time available for reproduction (Hanski *et al.* 2006).

The reason for immigration selecting for increased dispersal in the focal population is biased emigration: the dispersers and hence the immigrants are more dispersive than the average individual in the metapopulation and hence more dispersive than the average resident in the focal population. Note, however, that this applies on average; in particular populations the reverse may be true. Thus immigrants originating from relatively stable populations, which have a low frequency of fast-dispersing individuals on average, may have lower average dispersal rate than residents in small habitat patches, which have a high rate of population turnover and hence a high frequency of fast-dispersing individuals on average. The effect of extinction rate on dispersal evolution is due to the fact that the faster the populations go extinct the less time there is for local selection and emigration to reduce dispersal rate following the founder event. Our results for the Glanville fritillary support the effects of both the immigration rate and the extinction rate in increasing the frequency of fast-dispersing individuals in particular local populations.

Inbreeding is often thought to select for dispersal (e.g. Roze & Rousset 2005). Previous studies on the Glanville fritillary have shown that one generation of sib-mating is enough to lead to inbreeding depression that is strong enough (Haikola *et al.* 2001) to increase the risk of extinction of small local populations (Saccheri *et al.* 1998). However, females are not able to discriminate against siblings as mates (Haikola *et al.* 2004) and inbreeding can hardly explain the positive effect of immigration on dispersal. It is probable that the cognitive capacities of butterflies do not allow similar conditional dispersal decisions related to kin structure than observed in vertebrates.

### Habitat loss and the evolution of dispersal

Habitat loss and fragmentation alter the spatial structure and dynamics of populations, which influence the costs and benefits of dispersal and may therefore affect the evolution of dispersal. Whether habitat loss and fragmentation select for increased, decreased or non-monotonically changing rate of dispersal has been much debated (Ronce & Olivieri 2004; Hanski 2005). Given the multitude of factors affecting dispersal evolution it is not surprising that the evolutionary consequences of habitat loss and fragmentation may be complex.

**Figure 4:**
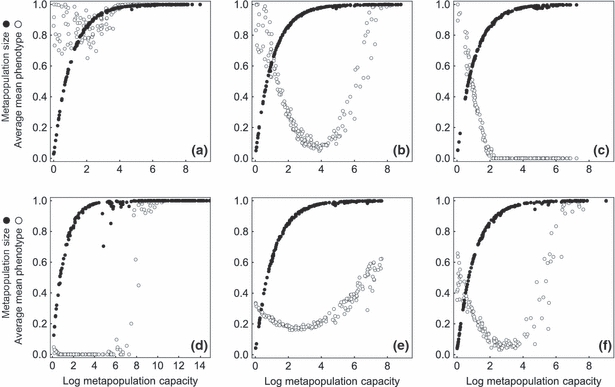
The equilibrium metapopulation size and the average frequency of fast-dispersing individuals in 200 patch networks with a dissimilar degree of fragmentation. Each dot represents one network. Each network has 100 patches with log-normally distributed areas, but the distributions were generated with different means and variances (exp (X), where X is the underlying normal distribution with mean and variance drawn from the uniform distributions [1..3] and [0..0.3], respectively). The amount and fragmentation of habitat in each network was measured by metapopulation capacity, and metapopulation size was measured as the weighted average of the patch occupancy probabilities as prescribed by the theory (Hanski & Ovaskainen 2000). Panels (a) to (c) depict three situations with decreasing strength of immigration in relation to the colonisation rate (*ρ*= 1, 0.5 and 0.1 in (a), (b) and (c), respectively; other parameter values are *r*_*0*_ = 1, *v*=1, *α* = 0.2, *γ* = 0, Δ = 5, *ε* = 0.01 and *c*=0.01). Panels (d) to (f) have the same parameter values as (a) except that there is less environmental stochasticity (*v*=0.5) in (d), there is local selection against fast-dispersing individuals (*γ* = 0.2) in (e), and dispersal rate is generally reduced (*c*=0.005) in (f).

The present results suggest one reason for the conflicting results. The long-term equilibrium rate of dispersal in habitat patch *i* depends on the sum of the immigration and extinction rates (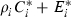; see Eq. 9), and as habitat loss and fragmentation may have opposing effects on these rates the overall effect depends on quantitative details. For instance, decreasing the areas of habitat patches generally increases extinction rates, because smaller populations have a higher risk of extinction, but decreases immigration rates, because smaller populations typically produce fewer dispersers. Changing the values of model parameters may therefore change the direction of selection due to habitat loss and fragmentation. Figure 4 gives the outcome of selection for six different combinations of parameters. It is apparent that, depending on the parameter values, average dispersal rate may decrease, increase or show a non-monotonic change with decreasing amount and increasing fragmentation of habitat (the amount of habitat decreases to the left in Fig. 4).

The above results provide insight to the likely evolutionary consequences of habitat loss and fragmentation. Given the high turnover rate in the Glanville fritillary metapopulation in the Åland Islands (Hanski 1999), one could expect that, in this case, habitat loss tends to increase dispersal rate. This is supported by the results of two different individual-based models that have been parameterized with empirical data (Heino & Hanski 2001; Zheng *et al.* 2009). That habitat loss and fragmentation increase dispersal rate in the Glanville fritillary is also supported by empirical data. Within the Åland Islands, dispersal rate is higher in the regions with lower density of habitat patches and lower frequency of patch occupancy (Zheng *et al.* 2009; Hanski 2011). At a larger scale, comparing the rate of dispersal in the fragmented landscape in the Åland Islands with that in a relatively continuous habitat in Xinjiang in China showed that dispersal rate was higher in the more fragmented landscape (Wang *et al.* 2011).

## Conclusion

We conclude by highlighting the contribution that the present model makes to the study of the evolution of dispersal. It is well known that mortality during dispersal selects against dispersal (Clobert *et al.* 2001; Ronce 2007), which effect is reflected in the present model by reduced immigration rate selecting against dispersal. Similarly, it is well known that environmental stochasticity that increases population fluctuations and the risk of extinction selects for dispersal by increasing the reproductive success of dispersers (Comins *et al.* 1980), which is reflected by high frequency of fast-dispersing individuals in newly-established populations in metapopulations (Hanski *et al.* 2006; Fig. 2 in this paper) and in marginal populations of species expanding their geographical ranges (Thomas *et al.* 2001). Finally, we would expect high frequency of fast-dispersing individuals among the immigrants to a particular population to increase the long-term average frequency of fast-dispersing individuals in that population, though we are not aware of any previous empirical studies demonstrating such an effect. The contribution of the present study is to put all these factors into the same model and to derive an expression for the long-term frequency of fast-dispersing individuals in different local populations in a patch network. Our results on *Pgi* polymorphism in the Glanville fritillary butterfly support the specific model predictions and thereby also the general notion that, in this case, the ecological and microevolutionary dynamics are closely coupled.
